# Frequency Modulation and Spatiotemporal Stability of the sCPG in Preterm Infants with RDS

**DOI:** 10.1155/2012/581538

**Published:** 2012-07-19

**Authors:** Steven M. Barlow, Mimi Burch, Lalit Venkatesan, Meredith Harold, Emily Zimmerman

**Affiliations:** Neuroscience, Human Biology, and Bioengineering, Department of Speech-Language-Hearing: Sciences and Disorders, Communication Neuroscience Laboratories, University of Kansas, 1315 Wakarusa Drive, Lawrence, KS 66049, USA

## Abstract

The nonnutritive suck (NNS) is an observable and accessible motor behavior which is often used to make inference about brain development and pre-feeding skill in preterm and term infants. The purpose of this study was to model NNS burst compression pressure dynamics in the frequency and time domain among two groups of preterm infants, including those with respiratory distress syndrome (RDS, *N* = 15) and 17 healthy controls. Digitized samples of NNS compression pressure waveforms recorded at a 1-week interval were collected 15 minutes prior to a scheduled feed. Regression analysis and ANOVA revealed that healthy preterm infants produced longer NNS bursts and the mean burst initiation cycle frequencies were higher when compared to the RDS group. Moreover, the initial 5 cycles of the NNS burst manifest a frequency modulated (FM) segment which is a significant feature of the suck central pattern generator (sCPG), and differentially expressed in healthy and RDS infants. The NNS burst structure revealed significantly lower spatiotemporal index values for control versus RDS preterm infants during FM, and provides additional information on the microstructure of the sCPG which may be used to gauge the developmental status and progression of oromotor control systems among these fragile infants.

## 1. Introduction

### 1.1. Suck Central Pattern Generator

The mammalian suck is the earliest-appearing somatic motor rhythm and is primarily controlled by a neural network known as the suck central pattern generator (sCPG). Suck appears *in utero* between 15 and 18 of weeks gestational age (GA) and the nonnutritive suck (NNS) is remarkably stable and well patterned by 32 weeks postmenstrual age (PMA) among healthy preterm infants [1]. The presence of a coordinated NNS is a good index of sucking skills, but not necessarily of an infant's readiness to orally feed [2]. Coordinated nutritive suck-swallow-breathe (bottle feeding) is usually not attained until 34 weeks PMA [2–4].

The sCPG consists of bilateral, linked internuncial circuits within the brainstem pontine and medullary reticular formation [5–7]. Based on animal models, the minimal circuitries for ororhythmic activity reside between the trigeminal motor nucleus and the facial nucleus in the brainstem [7] and are situated to function as premotor inputs to lower motor neurons. The sCPG is modulated by multiple inputs, including descending pathways from sensorimotor cortex and reciprocal connections with the cerebellum [8, 9], which serve to modulate ororhythmic activity. Thus, it is important to assist human infants to regulate their behavioral “state” through careful posturing and orientation during clinical testing as this will affect the nature of descending inputs to the sCPG. The sCPG can also be modified by sensory input arising from oral mechanoreceptors that encode the consequences of oral movements and external stimulation (e.g., breast, pacifier or bottle nipple, and touch) along central pathways of the trigeminal system. Suck entrainment has been demonstrated in term infants through 6 months of age using a patterned orocutaneous stimulus delivered to perioral and intraoral tissues [10]. Entrainment is defined as the phase locking of centrally generated suck motor patterns to an applied external stimulus, and represents a powerful method of achieving neural synchrony among sensorimotor pathways. Therefore, it is not surprising that stimulation of the lips and tongue is common method used to evoke sucking behaviors [11–13].

A precursor ororhythmic sensorimotor behavior involving a reduced set of orofacial muscle systems, nonnutritive suck (NNS) is typically observed in preterm infants between 28 and 33 of weeks gestation. The NNS is defined as a repetitive mouthing activity on a pacifier or a nipple without expelling any liquid stimulus [14, 15] and is thought to be regulated in part by a bilateral brainstem neural circuit known as the suck central pattern generator (sCPG) [5, 7, 16]. Wolff [15] described the NNS as changing across segments such that the first and second segments were faster than the third. Animal models suggest that the sCPG is sensitive to sensory input, modified by experience, and modulated by descending inputs from somatosensory cortex and reciprocal connections with the cerebellum. Sensitivity to punctate, low-amplitude high-velocity orocutaneous input to accelerate the development of NNS has recently been demonstrated in human preterm [17–19] and term infants [10].

Feeding readiness and inference to neural integrity is often evaluated by an infant's display of NNS and oromotor patterning [2, 20, 21]. The maturation and coordination of the NNS precedes the suck-swallow-breathe pattern associated with the slower 1 Hz pattern characteristic of the nutritive suck [22–24].

Suck is a precocial ororhythmic motor behavior in humans and is integral to competent oral feeds. However, premature infants often demonstrate oromotor dyscoordination and are unable to suck and feed orally [25, 26]. This represents a frequent and serious challenge both to the neonatal intensive care unit (NICU) survivors and the physician-provider-parent teams. The potential causes for delayed or impaired suck development are numerous and may result from neurologic insult to the developing brain, feeding intolerance, post-surgical recovery, diabetes, drug exposure, or as a result of ventilator interventions for delayed lung development or lung disease which interferes with ororhythmic pattern formation.

The interval of inactivity between NNS compression bursts is known as the “pause” period. Characteristics of the NNS can be described in terms of *coarse* and *fine* structure. The coarse structure of the NNS usually refers to a simple accounting of the number of suck bursts and pauses, respective duration, and the number and amplitude of suck compression cycles within each burst. The fine structure of the NNS defines within-burst characteristics such as the period and amplitude of each burst cycle [24] or a statistical measure of NNS burst pattern formation known as spatiotemporal stability [18, 19]. Precise measures of the coarse and fine structure of the NNS provide useful information correlated to developmental status and progression of oromotor control systems among these fragile infants [17, 18, 20, 27, 28].

The aim of the present study was to further explore the fine structure of the NNS burst using frequency and time domain measures, and exponential regression methods to capture the modulation inherent to NNS burst structure in health and disease. Such a model is likely to provide a new utility for comparison of oromotor skills across preterm groups varying in the degree of sensory deprivation/motor restriction due to O_2_ supplementation therapy and can be used to monitor change in sCPG pattern formation due to maturation and intervention. In the present paper, particular attention is given to the dynamics of NNS burst initiation and frequency modulation of suck compression cycles among RDS and healthy control preterm populations. We expect to find a relation between the NNS pattern formation and RDS severity which will lead to the formulation of a mathematical and statistical model of NNS burst cycle dynamics.

## 2. Methods

### 2.1. Participants

Participants included 32 preterm infants (16 F, 16 M) admitted and receiving care in the NICUs of Stormont-Vail HealthCare (Topeka, KS, USA) and the University of Kansas Medical Center (Kansas City, KS, USA). The mean gestational age (GA) was 30.61 weeks and the mean birth weight was 1424.15 grams. These infants were distributed among two groups including healthy control and RDS, according to their oxygen and percent daily oral feeding histories. At 34.11 weeks PMA, the control infants (*n* = 17) averaged less than a day and a half of oxygen therapy, no ventilation was required, and feeding 22.46% orally. Infants in the RDS group (*n* = 15) had an average of 34.2 days of oxygen therapy and demonstrated marked deficits in oral feeding, 3.87% PO (Table 1). All infants had been extubated for at least 5 days at the time of testing. Infants were consented with the additional inclusion criteria: head circumference within 10–90th percentile of mean for PMA, neurological examination showing no anomalies for PMA (response to light, sound, and spontaneous movements of all extremities), and with stable vital signs (heart rate, blood pressure, age appropriate respiratory rate, and oxygen saturation > 92 SpO_2_) to allow for NNS. Exclusion criteria were intracranial hemorrhage, hypoxia-ischemia encephalopathy, periventricular leukomalacia (PVL), neonatal seizures and culture positive sepsis or meningitis at time of testing, chromosomal anomalies, or craniofacial malformation.

### 2.2. Data Collection

Digital recording of NNS compression pressure waveforms was initiated after 32 weeks PMA and continued on a weekly basis until the infant was discharged or transferred from the hospital. Data considered in the present paper is based on two digitized samples, sampled at a 1-week interval of NNS compression pressure waveforms collected 15 minutes prior to a scheduled feed. Infants remained connected to their pulse-oximetry monitors during testing. The infant was placed in a developmentally supportive position, including head support, arms and hands swaddled at midline, background lighting dimmed, and facing the examiner to promote eye contact. The infant's pacifier was placed on a pressure-instrumented acetyl receiver specially designed to accommodate the Philips AVENT Soothie silicone pacifier. This configuration allowed real time sampling and analysis of the NNS using NeoSuck RT, a software program coded in our laboratory using C^++^. A 48′′ Luer pressure line coupled the pacifier-receiver handpiece to a calibrated Honeywell 5 psi pressure transducer. Transducer output was conditioned by a DC-coupled bridge amplifier (BioCom 215, Butterworth low pass @ 50 Hz). The infant-generated analog pacifier compression pressure signal was sampled in real time at 3 kHz (16-bits, National Instruments PCI-6062E).

Sampling of NNS behavior was initiated when the infant achieved an optimal behavioral state, that is, drowsy to active alert (state 3, 4, or 5 as described by the Naturalistic Observation of Newborn Behavior, Newborn Individualized Developmental Care and Assessment Program; NIDCAP) [29]. Three minutes of NNS behavior was digitized for each infant per session, with the most productive 2-minute epoch based on NNS cycle count subjected to formal quantitative and statistical analysis.

### 2.3. NNS Signal Analysis

**NNS Compression Waveform Discrimination**

**Figure 1:**
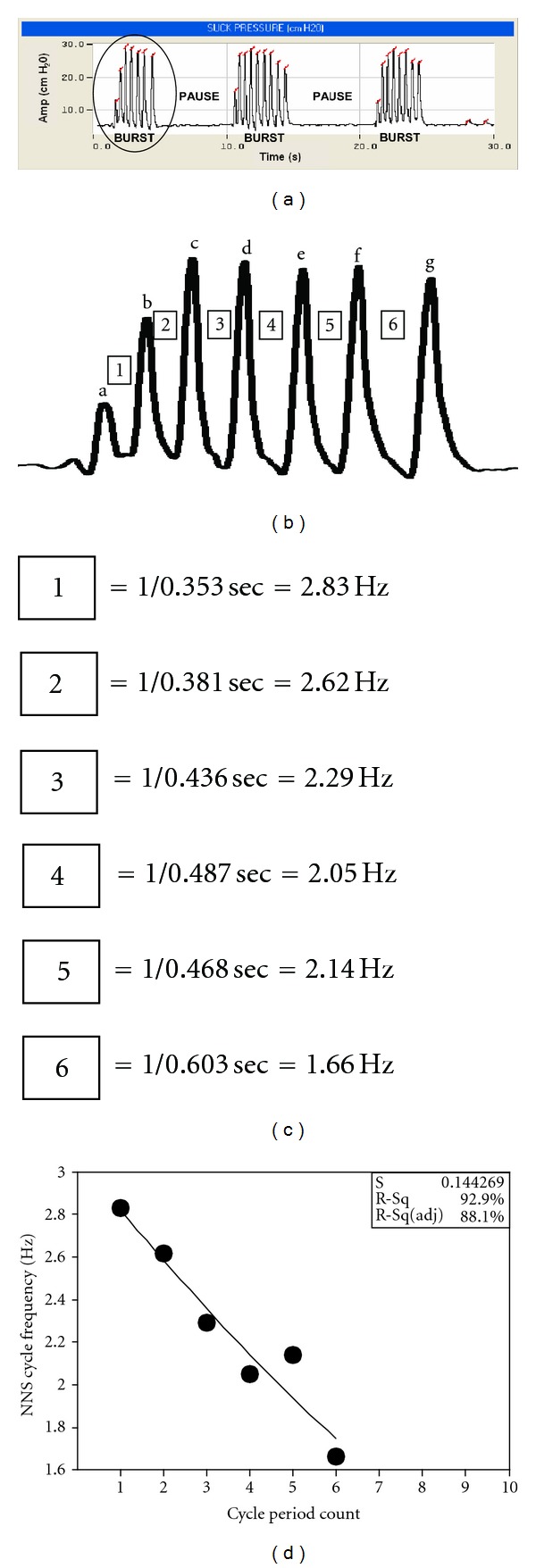
(a) Characteristic burst-pause pattern of NNS. (b) Outset window of the first burst in the 30-second sample. This burst has 7 cycles, identified by letters a–g, and 6 suck cycle periods, identified by numbers 1–6. (c) Individual cycle periods converted to frequency (1/*T*). (d) Plot of cycle period count versus suck cycle frequency (Hz) to demonstrate NNS frequency modulation (FM).

Nonnutritive compression suck cycle periods were obtained from the digitized NNS pressure waveform record obtained using a waveform discrimination and pressure threshold detection algorithm coded in the NeoSuck RT software program which automatically indexes pressure peaks for signals which exceed a preset 2 cm H_2_O pressure threshold. Operationally defined, an NNS burst in this context is defined as 2 or more NNS compression cycles having cycle periods of 1000 ms or less. Identification of the time-amplitude intercepts for individual pressure peaks is achieved by calculation of the first derivative of the pressure signal. A set of algorithms available in MatLAB are coded to automatically segment and index individual NNS compression cycles. The first derivative of the NNS pressure waveform is NNS pressure velocity. Pressure velocity “zero crossings” corresponds to the reversal in pressure trajectory associated with “peaks.” These peak indexes tallied as time-amplitude intercepts in the digitized NNS pressure record are coregistered with a pressure “history” or hysteresis function to identify major peaks from minor fluctuations in pressure slopes. This algorithm permits objective identification of NNS burst activity as distinct from nonorganized mouthing compressions. The resultant time period is converted to frequency using the formula *F* = 1/*T*; where *F* equals frequency (Hertz or Hz). This measure represents the instantaneous cycle rate between two consecutive pacifier nipple compression pressure peaks, and *T* is the time period (seconds) between two consecutive compression pressure peaks (Figure 1(b)).

### 2.4. NNS Spatiotemporal Index

The physiological approach to the assessment and habilitation of suck in the NICU includes a functional assessment of the integrity of the neural circuitry driving the sCPG through an analysis of suck pattern structure and stability [18]. Coordinated NNS that is minimally variable from burst-to-burst indicates motor system integrity and is an important foundation for coordination with other emergent behaviors, such as swallow and respiration. A highly promising digital signal processing technique known as the NonNutritive Suck Spatiotemporal Index (NNS STI) has been developed in our laboratory to quantify the emergence of stable NNS in preterm infants. The mathematical tenets underlying this computational technique have been used successfully to assess kinematic variability and pattern formation in limb [30, 31] and speech [32, 33] motor subsystems. The NNS STI provides the clinician with a single numerical value, calculated from the cumulative sum of the standard deviations on a set of five amplitude- and time-normalized suck pressure burst waveforms. The net statistic NNS STI represents the stability of the infant's oromotor sequence. For example, an NNS STI equal to 85 represents a highly variable NNS burst structure from one production to the next, whereas an NNS STI equal to 30 represents a relatively invariant, stable pattern of suck burst output. The NNS-STI is designed to quantify the infant's suck over a selected burst pattern epoch, thereby providing NICU clinicians with a summative index or “*gestalt*” of oromotor pattern formation and stability. Obtaining a two-minute sample of NNS behavior daily in the NICU with a physiological data acquisition microprocessor at cribside is sufficient to chart an infant's progress toward stable suck production [18, 19].

**Statistics and Nonlinear Modeling**

In order to model and quantify the frequency-modulated (FM) component of the NNS burst, the dependent measure of NNS cycle frequency will be regressed against NNS cycle index for each preterm group using an exponential decay model. NNS burst length will be compared between healthy preterm and RDS preterm infants using a repeated measures analysis of variance (SPSS). Each statistical measure will produce an *F*-score and is considered significant for *P-*values less than or equal to 0.05.

## 3. Results

An example of the basic NNS burst-pause structure sampled from a healthy preterm infant at 34 weeks PMA is shown in Figure 1(a). The initial phase (2-3 cycles) of the burst shows a rapid growth in compression cycle amplitude followed by a slower amplitude decay phase for each of the 3 bursts in this sample. An expanded view of the first NNS burst is shown as an outset pressure waveform in Figure 1(b). In addition to the amplitude modulation (AM), there is evidence of frequency modulation (FM) in Figure 1(c), with the interpeak time intervals translating to a steady decline in cycle frequency from 2.83 Hz (period 1) to 1.66 Hz (period 2). A quadratic regression shows a significant negative relation between NNS cycle frequency and cycle period count with an *R* ^2^ of 92.9% for this NNS burst (Figure 1(d)).

**Figure 2:**
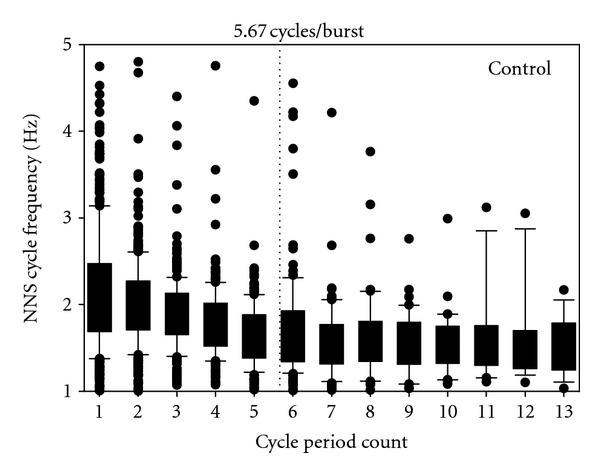
Boxplot of NNS cycle frequency (Hz) as a function of NNS cycle period count for control preterm infants (*N* = 17). Mean NNS burst length is indicated by dotted vertical line.

**Figure 3:**
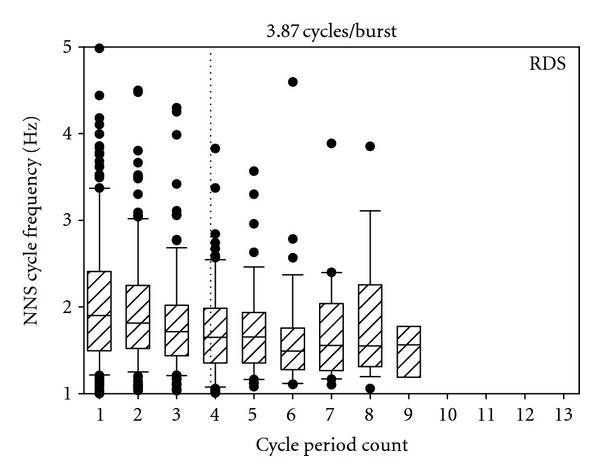
Boxplot of NNS cycle frequency (Hz) as a function of NNS cycle period count for RDS preterm infants (*N* = 17). Mean NNS burst length is indicated by dotted vertical line.

**Figure 4:**
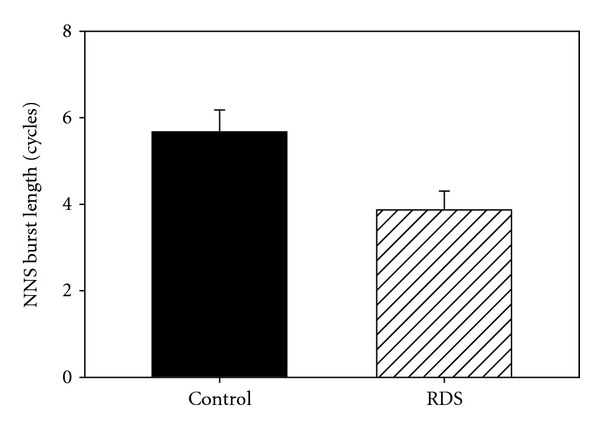
Bargraph of NNS burst length (cycles) for control and RDS preterm infants.

The distribution of NNS compression cycle period frequencies is shown for control (Figure 2) and RDS infants (Figure 3). The analyzed data includes a total of 400 NNS bursts (control = 234; RDS = 166), and a boxplot of NNS cycle frequency by period count for each group generally follows a negative exponential decay function. The mean period frequency in each plot is represented by the dotted vertical line. ANOVA revealed a significant difference (*F*(1,398) = 25.63, *P* < 0.0001) in the mean burst length between the groups with the longest mean burst length (5.67 cycles/burst) belonging to the healthy preterm controls and the shortest mean burst length (3.87 cycles/burst) associated with the RDS infants (Figure 4).

**Figure 5:**
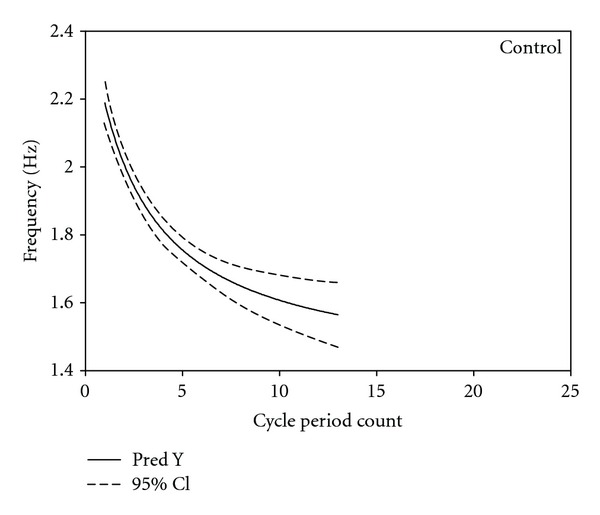
Exponential regression decay function for control NNS burst cycle period frequency by NNS cycle period count. 95% confidence interval given by the dotted line.

**Figure 6:**
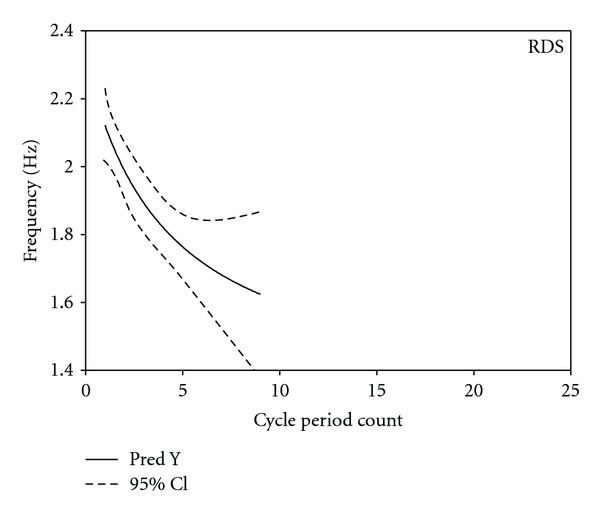
Exponential regression decay function for RDS NNS burst cycle period frequency by NNS cycle period count. 95% confidence interval given by the dotted line.

Exponential decay regression analyses for NNS burst cycle frequency as a function of cycle period count revealed highly significant negative decay functions for both control and RDS infants. The predicted-*Y* and 95% confidence intervals (CI) for control and RDS NNS cycle frequency are shown in Figures 5 and 6, respectively. The predicted-*Y* for healthy control NNS burst cycle frequency was described by the equation *Y* = 1.3902^1.9166/*x*+3.2240^ (*F* = 86.06, *P* < 0.0001), and the predicted-*Y* for RDS NNS burst frequency was described by the equation *Y* = 1.3082^3.1253/*x*+5.4661^ (*F* = 11.80, *P* < 0.0001), where *Y* is the NNS cycle frequency and *X* is the NNS cycle period count. Overall, the variability in NNS cycle frequency is less among control compared to RDS infants as shown by the 95% CIs. As expected, maximum NNS burst length was longer for control (13) versus RDS (9) infants.

**Figure 7:**
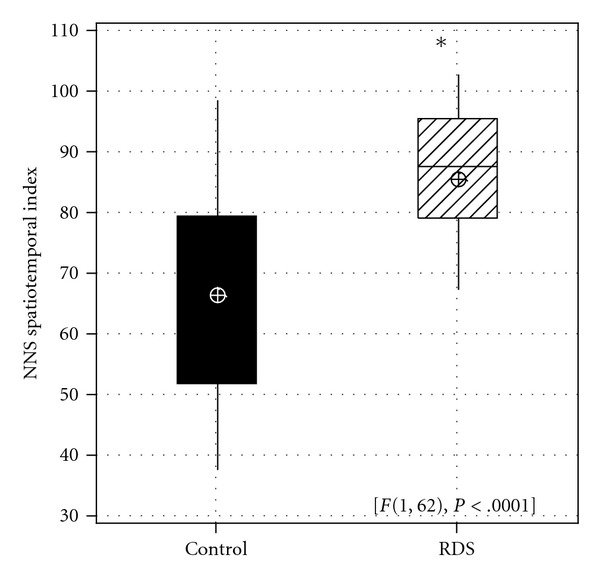
Boxplot of nonnutritive suck spatiotemporal index (NNS STI) for control and RDS preterm infants.

The motor gestalt of NNS burst pattern form, quantified as the NNS spatiotemporal index was significantly lower for healthy control (STI = 66.29) versus RDS (STI = 85.44) preterm infants (*F*(1,62) = 24.44, *P* < 0.0001) (Figure 7). The lower STI observed among control infants reflects a more developed, less variant NNS burst compression pressure pattern compared to their RDS counterparts.

## 4. Discussion

By 32 weeks PMA, a well-organized NNS burst in a healthy infant consists of approximately 7 cycles at an average frequency of 2 Hz, and a mean peak compression pressure of 17 cm H_2_O [4, 15, 27, 28]. The present study has detailed a “fine” feature of the NNS burst in healthy preterm infants at 34 weeks PMA, described as an FM or frequency modulated component of NNS burst pattern formation which exhibits a relatively invariant profile with an initial period frequency of 2.2 Hz that decays exponentially to approximately 1.6 Hz by cycle period number 13. This stable pattern of FM modulation exhibited among the 17 healthy preterm controls at 34.11 wks PMA was markedly different for preterm RDS infants who endured more than a month of O_2_ supplementation therapy (mean = 34.2 days). For the RDS profile, the FM feature is more variable as evidenced by the spread of the 95% confidence intervals and begins at a lower start frequency, with exponential decay over a shorter maximum NNS burst length of 9 cycle periods of 2.2 Hz.

We attribute the reduced NNS burst structure manifest among the sample of 15 RDS infants to an altered and maladaptive experiential set during a critical period of assembly for the central pattern generation and refinement of suck. This includes both sensory deprivation and motor restriction of the orofacial apparatus which contribute to developmental delay in the integrity of the sCPG, ultimately contributing to the delays observed in attainment of independent oral feedings [17]. Development of the central nervous system, including central pattern generators can be modified by environmental factors [27, 28, 34–37]. Lengthy oxygen supplementation procedures in the NICU cost the preterm infant precious sensory and motor experiences during a critical period of brain development when the central patterning of suck and prefeeding skills are being refined. Even the presence of a nasogastric (NG) feeding tube has negative effects on sucking and breathing [38]. Trussing the lower face with poly tubes and tape also restricts the range and type of oral movements and limits cutaneous experiences with the hand and fingers. Interruption of these experiences may impair fragile syntheses of how the brain maps these functions [39, 40]. For some preterm infants, poor suck and oromotor dyscoordination persists well into early childhood and may lead to significant delays in the emergence of other oromotor behaviors, including feeding, babbling, and speech-language production [41, 42]. Failure to establish oral feeding skills in the NICU may result in the infant being sent home on gavage or gastric tube feedings, and hinder the development of oral feeding behaviors. The difficulties associated with establishing oral feed competence along with the additional costs for extended hospitalization underscore the need for precise assessment and therapeutic tools to facilitate the development of normal oral motor skills [11, 12, 43, 44].

Establishing a patterned NNS for the developing infant carries many positive benefits, including growth, maturation, and gastric motility, while decreasing stress [24, 43–49], improving state control before-feeding [45, 47, 50–52] and after-feeding [48], and enhancing oral feeds [17–19, 53]. Use of a pacifier for NNS appears to decrease the frequency of apnea and cyanosis, and improve breastfeeding scores [54]. The NNS accelerates the transition from tube to independent oral feeding and is presumed to enhance the maturation of neural systems responsible for ororhythmic activity [55–57]. The sensory consequences associated with the production of NNS appear to provide beneficial effects on oral feeding performance and the development of specific sucking skills [11, 12]. Accurate assessment of oromotor dyscoordination in the preterm infant extends beyond the immediate issues surrounding the transition to oral feed competency, and may serve as a potent clinical marker for brain development and neurodevelopmental outcomes [20].

**Modulation of Biological Rhythms**

Frequency modulation of a motor output can result for a number of reasons including metabolic demands, cellular mechanisms, or recruitment of cells. Metabolic demands can force a change in frequency. For example, in heart or respiratory rate, modulation in frequency results when metabolic demands change. An increase in the metabolic demands of the body during exercise results in an increase in the heart and respiratory rates. Related to the modulation of CPGs, Grillner [58] identified the underlying cellular mechanisms involved in these neural circuits to include reciprocal inhibition, mutual excitation, plateau properties, or spike frequency adaptation (Ca^++^-dependent K^+^ channels). Frequency modulation could be the result of interplay between these cellular mechanisms resulting in a fine-tuned and consistent output so that each burst starts with higher frequency cycles and then decreases as the burst progresses.

Frequency modulation of a motor pattern may also occur as part of a sensory feedback loop providing the sCPG with information about the phase of the motor behavior. For example, in animal studies of gait, the change in frequency was the result of peripheral afferent feedback during both ordinary gait and in tasks that required modulation of the gait [59]. Grillner and Zangger [60] also discovered that a motor pattern could breakdown in the absence of sensory input.

**Significance of Frequency Modulation in a Biological System**

Physiological systems that typically demonstrate modulation are considered disordered when modulation is diminished or absent [61]. This understanding provides a basis for determining the degree of average modulation in NNS and the extent to which the modulation is influenced by environmental factors, genetic defects, or damage to the central nervous system (CNS). Exploration of knowledge in this area would then provide insight into deficits and potential diagnostic and intervention methods. Animal studies have explored the effects of lesions in different locations of the CNS. For example, motor patterns produced by decerebrate and decorticate cats highlighted the ability to produce rhythmic behavior in both models, but the inability to modulate in response to the environment or to specific needs of the animal when the lesion eliminated input from the basal ganglia is yet to be highlighted [62]. Understanding more about the components of the sCPG could identify a CNS location that is responsible for the temporal feature of the FM NNS.

## 5. Conclusion

In summary, healthy preterm infants manifest a significantly longer NNS burst structure when compared to infants with RDS. Second, there is an FM feature of NNS burst formation that is distinctly different for healthy control and RDS infants. Third, healthy preterm infants suck at a higher frequency at the onset of the suck burst when compared with the RDS infants. Finally, for both infant groups, the suck cycle periods increase in duration from burst onset to completion according to an exponential decay function. The ability of practitioners in the NICU to rapidly quantify both the coarse and fine features of NNS is expected to lead to more efficient, physiologically guided interventions to allow the preterm infant to safely advance to independent oral feeding.

Future studies could explore the FM NNS and burst evolution as a result of external stimulation. Such stimulation might include presentation of a pacifier that emulates the NNS in its FM burst-pause characteristics. Such information may be used as a diagnostic tool for identifying aberrant NNS patterns and provide status information on CNS organization in preterm-term populations that have experienced insults such as intraventricular hemorrhage (IVH), mild-moderate white matter injuries (PVL), chronic lung disease, infants of diabetic mothers, infants recovering from cardiac surgery, or genetic anomalies such as Down's syndrome.

## Figures and Tables

**Table 1:** 

Variable	Control (*N* = 17)	RDS (*N* = 15)
SEX (male : female)	8 : 9	8 : 7
Birth GA (wks)	31.5 (1.4)	29.6 (1.3)
Birth weight (gms)	1518.7 (318.6)	1317.0 (480.8)
PMA (wks)		
Session 1	33.56 (1.7)	34.69 (1.9)
Session 2	34.66 (1.6)	35.41 (1.8)
Mean	**34.11 (1.7)**	**35.05 (1.9)**
% Oral feed		
Session 1	13.2 (4.0)	1.73 (6.4)
Session 2	34.47 (9.5)	6.00 (10.1)
Mean	**22.46 (6.8)**	**3.87 (8.6)**
O_2_ history (days)		
VENT	0.00 (0.0)	5.73 (8.1)
CPAP	0.71 (1.1)	8.40 (8.6)
Cannula	0.65 (1.3)	20.07 (18.9)
Total	**1.35 (1.7)**	**34.20 (28.6)**
